# Regulatory systems for gene expression control in cyanobacteria

**DOI:** 10.1007/s00253-019-10344-w

**Published:** 2020-01-22

**Authors:** Petra Till, Jörg Toepel, Bruno Bühler, Robert L. Mach, Astrid R. Mach-Aigner

**Affiliations:** 1 Christian Doppler Laboratory for Optimized Expression of Carbohydrate-Active Enzymes, Institute of Chemical, Environmental and Bioscience Engineering, TU Wien, Gumpendorfer Str. 1a, A-1060 Vienna, Austria; 2 Institute of Chemical, Environmental and Bioscience Engineering, TU Wien, Gumpendorfer Str. 1a, A-1060 Vienna, Austria; 3 Department of Solar Materials, Helmholtz-Centre for Environmental Research GmbH—UFZ, Permoserstrasse 15, 04318 Leipzig, Germany

**Keywords:** Cyanobacteria, Gene expression, Induction systems, Promoters, Riboswitches, Riboregulators, Regulatory circuits

## Abstract

As photosynthetic microbes, cyanobacteria are attractive hosts for the production of high-value molecules from CO_2_ and light. Strategies for genetic engineering and tightly controlled gene expression are essential for the biotechnological application of these organisms. Numerous heterologous or native promoter systems were used for constitutive and inducible expression, yet many of them suffer either from leakiness or from a low expression output. Anyway, in recent years, existing systems have been improved and new promoters have been discovered or engineered for cyanobacteria. Moreover, alternative tools and strategies for expression control such as riboswitches, riboregulators or genetic circuits have been developed. In this mini-review, we provide a broad overview on the different tools and approaches for the regulation of gene expression in cyanobacteria and explain their advantages and disadvantages.

## Introduction

Cyanobacteria are a very diverse group of photosynthetic microorganisms. They colonise all light-exposed habitats on Earth, including marine water, freshwater, soil, glaciers, deserts and hot springs (Whitton and Potts 2000). The phylum is divided into five subsections and comprises unicellular and filamentous strains (Rippka et al. 1979). Some of the latter are capable to differentiate some vegetative cells into specialized cell types (i.e., heterocysts) (Stewart et al. 1969).

As photosynthetic microorganisms, cyanobacteria are appealing models for studying photosynthetic processes on a single-cell level and aspired hosts for the large-scale production of high-value molecules in industry (Abed et al. 2009). Despite their high potential in these fields, strategies for genetic engineering and tightly controlled gene expression still lag behind the tools available for common heterologous hosts. Methods for classical mutagenesis are well established in model cyanobacteria (Grigorieva and Shestakov 1982; Marraccini et al. 1993) and also the Clustered Regularly Interspaced Short Palindromic Repeats (CRISPR)-technology has successfully been applied in these organisms (Ungerer and Pakrasi 2016; Wendt et al. 2016). However, the application of these methods is limited by the toxicity of Cas9 (Wendt et al. 2016), the high ploidy level of many cyanobacteria (Griese et al. 2011) and the varying efficiency in the diverse cyanobacterial strains. Another obstacle in genetic engineering is the lack of regulatory elements and induction systems that can be precisely controlled. Several commonly used heterologous promoters are substantially leaky or perform poorly in cyanobacteria (Guerrero et al. 2012; Huang et al. 2010). As an example, expression levels obtained from Ptrc (a hybrid of the *trp* and *lac* promoters from *E. coli*) are nearly equal in the presence or absence of the inducer. Native promoter systems in contrast often allow relatively tight induction control, but usually yield lower expression levels and depend on inducers that regulate and thus interfere with endogenous metabolic processes in cyanobacteria (Guerrero et al. 2012; Huang et al. 2010). Reasons for the respective technical restrictions are the differences in gene expression control in heterotrophic hosts compared to cyanobacteria and the limited knowledge on the underlying mechanisms in the latter. Anyway, in the last years research in this field has been intensified, some existing promoter systems could be improved and novel promising promoters were engineered for the use in cyanobacteria. Moreover, RNA-based tools such as riboswitches, riboregulators or small RNAs (sRNAs) as well as genetic circuits emerged as highly promising strategies for gene expression control in cyanobacteria (Higo et al. 2017, 2018b; Ma et al. 2014; Taton et al. 2017; Ueno et al. 2018). Furthermore, striking improvements of gene expression could be achieved by alteration of regulatory elements such as the ribosome binding site (RBS) (Englund et al. 2016; Thiel et al. 2018; Wang et al. 2018). Different tools for RBS design are available, such as the Ribosome Binding Site Calculator (Salis 2011), the RBS Designer (Na and Lee 2010) or the UTR Designer (Seo et al. 2013). More details on the importance of RBS choice and engineering are provided elsewhere (Immethun and Moon 2018; Sun et al. 2018a). In this mini-review, we focus on promoters, riboswitches, riboregulators and genetic circuits. The knowledge on traditionally used systems is briefly summarised and current findings and novel tools are more accurately discussed. Details on all regulatory tools are provided in Online Resource 1. Abbreviations of the discussed cyanobacterial strains and short information about them are provided in Table 1.

**Table 1:** Cyanobacterial strains

Strain	Abbreviation	Notes
*Anabaena* sp. PCC 7120	A_7120	Model filamentous, heterocyst-forming cyanobacterium
*Anabaena variabilis* ATCC 29413	Av_29413	Model filamentous, heterocyst-forming cyanobacterium
*Chroococcidiopsis*	–	Ancient coccoidal cyanobacterium (Imre Friedmann and Ocampo-Friedmann 1995)
*Leptolyngbya* sp. strain BL0902	L_BL0902	Filamentous, grows well in outdoor bioreactors
*Nostoc punctiforme* ATCC 29133	Np_29133	Filamentous, heterocyst-forming cyanobacterium
*Spirulina platensis* strain C1	Sp_C1	Planktonic filamentous cyanobacterium
*Synechocystis* sp. PCC 6803	Sy_6803	Model unicellular cyanobacterium
*Synechocystis* sp. strain PCC 6714	Sy_6714	Closely related to Sy_6803
*Synechocystis* sp. strain ATCC27184	Sy 27184	Glucose-tolerant Sy_6803
*Synechocystis* sp. strain WHSyn	Sy_WHSyn	Unicellular cyanobacterium, capable of grow in a wide range of salinities
*Synechococcus* sp. strain PCC 73109	Sc_73109	Closely related to Sce_7002
*Synechococcus elongatus* PCC 6301	Sce_6301	Freshwater unicellular cyanobacterium
*Synechococcus elongatus* PCC 7942	Sce_7942	Model freshwater unicellular cyanobacterium, formerly named *Anacystis nidulans* R2
*Synechococcus elongatus* PCC 7002	Sce_7002	Model marine unicellular cyanobacterium, fast growing
*Synechococcus elongatus* UTEX 2973	Sce_UTEX	Unicellular cyanobacterium, rapid autotrophic growth

## Promoters

The traditionally used regulatory elements for the control of gene expression are promoters. It can be distinguished between constitutive and inducible promoters. The latter are needed when temporary expression of the target gene is required, such as for the expression of gene products that are lethal for the cell or for components that are sensitive to certain conditions (e.g. O_2_ or light). Besides the regulatory mode, the promoters applied in cyanobacteria can be distinguished based on their origin. Commonly used heterologous promoters from *E. coli* or bacteriophages, native/endogenous promoters and synthetic or hybrid promoters have been employed. Crucial characteristics of such promoters are the expression strength, the induction control (i.e. baseline expression under non-inducing conditions) and the dynamic range (ratio between expression levels under inducing and non-inducing conditions). A comprehensive list of the promoters used in cyanobacteria and information on their properties are provided in Online Resource 1. However, it has to be considered that a comparison of the parameters derived from different studies has to be treated with caution. The output reported for the respective expression system depends on several factors, such as the applied substrain (as reported for Sy_6803; Zavřel et al. 2017), the type of gene expressed, the quantification method, the cultivation conditions, the reference value for calculations and the interplay of different regulatory elements.

### Constitutive promoters

Most constitutive promoters commonly used in cyanobacteria are native promoters, such as P*cpc* or P*psbA*. Both promoters control the expression of main components of the photosynthetic apparatus and thus are strong and present in nearly all cyanobacteria. **P*psbA*** promotes transcription of the gene encoding the D1 protein of photosystem II (Mohamed and Jansson 1989). It actually is induced by light, but has been widely used as a constitutive expression system under constant light conditions. **P*cpc*** controls the expression of the *cpc* operon, which encodes phycocyanin, a pigment protein of the light-harvesting complex (i.e. phycobilisome) (Johnson et al. 1988). The *cpc* operon encodes several subunits of the phycobilisome as well as associated linker proteins. Often the first gene in the operon is *cpcB*. Thus, the promoter is also commonly referred to as P*cpcB*. Native and slightly modified variants of P*cpc* (e.g. **P*cpcB***, **P*cpc*560**, **Pcpt**, **P**_**CP**_) from Sy_6803, Sy_6714 and Sp_C1 have been successfully applied in Sce_7942, Sy_6803 and Sce_7002 (Imashimizu et al. 2003; Jeamton et al. 2011; Markley et al. 2015; Xu et al. 2011; Zess et al. 2016; Zhou et al. 2014). Particularly high product yields (15% of total soluble protein) were achieved by the application of a truncated version of P*cpcB* from Sy_6803, termed P*cpc*560 (Zhou et al. 2014). In contrast to this finding, Wang and co-workers recently reported a very poor performance of this promoter (14% activity of Ptrc) and other P*cpc* variants (Wang et al. 2018). They analysed the usability of 17 native and heterologous hybrid promoters for the production of the ethylene-forming enzyme (EFE) in Sy_6803. Besides P*cpc* variants, also different variants of P*psbA2* from Sy-6803 and P*psbA* from the flowering plant *Amaranthus hybridus* were employed and yielded moderate to high expression levels. The best results (up to 12.6% of total soluble protein) were obtained from PpsbA*, a hybrid version of P*psbA* from *Amaranthus hybridus* and the Cu^2+^ inducible promoter P*petE* from Sy_6803 (Wang et al. 2018). Good performance of P*psbA* has also been reported in other cyanobacterial strains such as A_7120 (Elhai 1993), Sce_7942 (formerly *Anacystis nidulans* R2) (Dzelzkalns et al. 1984), Sce_7002 (Jacobsen and Frigaard 2014) and *Chroococcidiopsis* (Billi et al. 2001). **P*psbA2*** from Sy_6803 has been widely used as a native system in this strain. Recently, improvement of the strength of this promoter was achieved by the application of a truncated version, termed **P*psbA2*S** (Englund et al. 2016). Lots of other native promoters controlling the expression of photosystem II such as **P*psbA1*** from Sce_7942 (Taton et al. 2014), P*psbA1* from A_7120 (Chaurasia and Apte 2011) or **P*psbAIII*** from S_7942 (Li and Golden 1993) as well as heterologous P*psbA* from the pea plant (Ungerer et al. 2012) have been applied in synthetic biology approaches. However, most of these promoters are not or only slightly induced in response to varying light conditions. For details refer to the ‘Native, inducible promoters’ section and Online Resource 1.

Another native promoter commonly used in cyanobacteria is the RuBisCO promoter **P*rbc*** (or P*rbcL*). Variants of P*rbc* originating from different cyanobacteria, i.e. A_7120 (Elhai 1993), Sce_6301 (Takeshima et al. 1994), Sce_7002 (Ruffing et al. 2016), Sce_7942 (Deng and Coleman 1999) and Sy_6803 (Huang et al. 2010), have been employed. Comparative promoter studies revealed a low to moderate expression strength of P*rbc* relative to other promoters such as P*psbA* and promoters of the J23-series described below (Englund et al. 2016) or the heterologous hybrid promoter P*trc*1O (Huang et al. 2010). Also, Ruffing and co-workers reported a low activity of native P*rbc* in Sce_7002 relative to other endogenous promoters (Ruffing et al. 2016). They compared Ypet reporter gene production from 25 uncharacterised native promoter systems. The highest expression levels were obtained for **P*A2520*** and **P*A2579*** during the stationary growth phase. Similarly, nine novel native promoters have recently been characterised and compared to well-known expression systems (Liu and Pakrasi 2018). **P*sll1626*** was identified as the most promising candidate, yielding expression levels approximately 5-fold higher than P*rbc*. Another well-studied, weakly constitutive promoter is **P*rnpB*** from Sy_6803, which mediates transcription of the gene encoding the RNA subunit of RNase P. Expression levels are even lower than those obtained from P*rbc* (Englund et al. 2016; Huang et al. 2010). Such promoters are suitable for low-level expression of repressors involved in regulatory circuits such as LacI or TetR (see following sections), but are not applicable for enzyme (over)production.

Besides native constitutive promoters, some strong synthetic and hybrid promoter systems have been developed for applications in cyanobacteria. The most prominent systems are the artificial BioBrick promoters from the **J23-series** (BBa_J23100–BBa_J23119) from the iGEM Registry of Standard Biological Parts (Camsund et al. 2014). In Sy_6803, the **J23119 promoter** was found to be the strongest and has successfully been applied in several cyanobacteria including Sce_7002 (Markley et al. 2015), Sce_7942 (Huang et al. 2016), Sce_UTEX (Ungerer and Pakrasi 2016) and A_7120 (Higo et al. 2018a). But also the activity of several other J23-promoters has been demonstrated in Sy_6803 and Sce_7942.

Semisynthetic **PconII** constitutes another strong promoter. Like the J23119 promoter, PconII can be applied in a broad range of cyanobacterial strains, including Sce_7942, Sy_6803, Sy_WHSyn, A_7120 and L_BL0902 (Ma et al. 2014; Taton et al. 2014). High expression levels (higher than from P*psbA1*) were obtained for all tested strains except for A_7120. Here, PconII showed only moderate performance.

Also, **P**_**R**_**-P**_**S**_ was emphasised as a highly efficient expression system, producing yields of up to 15% of total protein in Sce_7942 (Chungjatupornchai and Fa-aroonsawat 2014). The promoter was created by fusion of a truncated variant of P_R_ from Sce_7942 and P_S_ from *E. coli* and is one of the strongest expression systems in cyanobacteria.

For any constitutive or inducible promoter, expression may be improved via a coupling to the T7 RNA polymerase. For this purpose, the target gene is expressed under the control of the **T7 promoter** from *E. coli* phage λ, while the expression of the T7 RNA polymerase is controlled by the desired constitutive or inducible promoter. Using this system, a 10-fold higher expression yield compared to direct expression from the constitutive or inducible promoter was achieved in A_7120 (Wolk et al. 1993).

Moreover, several, especially IPTG-regulated, heterologous expression systems such as Ptrc are poorly regulated in cyanobacteria by their inducer. They, however, enable high expression levels. Hence, these promoters have also been applied for constitutive expression in cyanobacteria (Xiao et al. 2018). These promoters will be discussed in more detail in the next section. Anyway, the most efficient constitutive expression systems reported for cyanobacteria are PpsbA*, P*cpc*560 and P_R_-P_S_, followed by J23119, PconII and other *psbA* promoters. Studies on the performance of some of these promoters, especially P*cpc*560, however are inconsistent.

### Heterologous, inducible promoters

Promoters commonly used in *E. coli* such as P*lac*, Ptrc, P*tet* or the temperature-dependent cI-repressed P_R_ promoter from *E. coli* phage λ do not perform well in cyanobacteria (Huang et al. 2010). Their application results in poor or no transcription or non-adequate induction.

Most of the heterologous, poor performing promoters originate from the *E. coli* induction systems of the *lac* operon (based on **P*lac*)**. The lac system is based on induction by allolactose or its synthetic, metabolism-independent analogue IPTG and suppression by the separately encoded Lac-repressor LacI (Gilbert and Müller-Hill 1966). P*lac*-derived promoters can contain either one or two lac operators and encompass P*lac* itself and several hybrid versions, especially P*trp*-P*lac* hybrids such as **Ptac**, **Ptic**, **Ptrc** (or Ptrc1O), **Ptrc2O** and **P*trp*-P*lac*** (Brosius et al. 1985; Huang et al. 2010; Niederholtmeyer et al. 2010). Most of the P*trp*-P*lac* hybrids show a high similarity. Ptac, Ptrc and Ptic only differ in terms of the interspace length between the −35 and the −10 region (16, 17 and 18 bp, respectively) (Brosius et al. 1985). The shorter versions were slightly more active than Ptic. In contrast to this, in a more comprehensive study by Albers and co-workers, the highest activities were observed for the longer variants Ptrc and Ptic (Albers et al. 2015). This trend is well consistent with recent findings for PpsbA*, thus indicating that 17–18 bp is the optimal interspace length in cyanobacteria (Wang et al. 2018). Another crucial aspect for the quality of P*lac*-derived promoters is the number of *lac* operators. For Ptrc and Ptac, which contain only one *lac* operator, usually a moderate to high promoter strength was reported (even higher than for P*psbA* (Wang et al. 2018)). They, however, feature dramatically high baseline expression (leakiness) in the absence of the inducer in Sy_6803, Sy_27,184, Sce_7942 and A_7120 (Elhai 1993; Geerts et al. 1995; Guerrero et al. 2012; Huang et al. 2010; Huang and Lindblad 2013). In contrast to this, the variant Ptrc2O, which contains two operator sequences, is tightly repressed by LacI, but achieves only low expression levels upon induction with IPTG (Huang et al. 2010). Similarly, efficient control was observed for a Ptic-based promoter containing two lac-operators referred to as **Psca6-2**. Besides **Psca3-2** (a Ptac-based version), this promoter was reported as strong in relation to other investigated Ptic/Ptac-derived variants, but it was not compared to other promoter systems such as P*psbA* (Albers et al. 2015). Furthermore, the hybrid promoter **PA1*lac*O-1** was reported to be a promising candidate, but results regarding leakiness are inconsistent (Camsund et al. 2014; Guerrero et al. 2012). Finally, the problem of leakiness versus low activity of IPTG-induced systems could not be solved. However, promoters such as Ptrc or Ptrc2O might be useful tools as constitutive expression systems as they are highly active in the absence of the LacI repressor (Huang et al. 2010).

Similar to the IPTG/LacI-based system, the *E. coli*-derived induction system of the tetracycline-resistance operon *TN10* (i.e. **P*tet***) is controlled by a chemical inducer and a transcription factor, i.e. anhydrotetracyline (aTc, a non-toxic analogue of tetracycline) and TetR, respectively. Compared to IPTG-induced systems, a good performance can be achieved using Pt*et*-derived expression systems optimised for cyanobacteria. However, applications are challenged by the photolability of aTc (Huang and Lindblad 2013; Zess et al. 2016). For P*tet* itself, inconsistent results were achieved in different cyanobacterial strains. In Sy_6803, the activity of P*tet* was low and insufficiently repressed in the absence of the inducer (Huang et al. 2010; Immethun et al. 2016), whereas in A_7120 recently a tight expression control was reported, resulting in a 40-fold dynamic range (Xiao et al. 2018). However, the promoter strength was very low in this strain (i.e. 0.5% activity of Ptrc). High expression levels in contrast were achieved in Sce_7942 (i.e. 50% activity of Ptrc), but expression was also observed in the absence of aTc (Kim et al. 2017). The performance of P*tet* was strikingly improved by mutation of the 5′-GGG-3′ located immediately downstream of the −10 element on the non-template strand to 5′-GGC-3′, resulting in the **L03 promoter** (Huang and Lindblad 2013). In Sy_27,184 (i.e. glucose-tolerant Sy_6803), this promoter achieved high expression levels, good repression in the absence of aTc and a 290-fold dynamic range. Even strikingly higher dynamic ranges (i.e. 1200-fold and 18,000-fold) for the L03 promoter were obtained in A_7120 by the expression of *tetR* under the control of the nitrate-specific P*nirA* promoter (Higo et al. 2016, 2017). In Sy_6803, in contrast, Yao and co-workers reported also a high activity for L03 promoter, but leaky expression in the absence of the inducer (Yao et al. 2016). Differences in the dynamic range and leakiness of aTc-induced systems might be explained by strain-specific effects or by the promoter chosen for the expression of *tetR*. An impact of repressor levels on the final output of the induced promoter was also reported for lac-derived systems (Camsund et al. 2014).

Both aTc- and IPTG-based induction systems were combined with the strong constitutive *cpcB* promoter by synthetic fusion of P*cpcB* variants with the *lac* or *tet* operators, with the goal to produce strong, inducible expression systems. **PEZ*tet***, the hybrid of P*cpcB* and two *tet* operators, showed moderate expression and tight control of induction, resulting in a 32-fold dynamic range (Zess et al. 2016). The performance of Pcpt-*lac* operator hybrids was even better. Best results were achieved for **PcptOO-cLac143**, which is featured by high promoter strength, low background expression and a 48-fold dynamic range (Markley et al. 2015). Further hybrids of either of the two repression systems with the P_L_ promoter from *E. coli* phage λ, termed **PL*lac*O1** and **PL*tet*O-1**, yielded rather poor promoter strengths and induction control (Huang et al. 2010; Oliver et al. 2013).

Three heterologous, metabolite-based promoters which have recently been engineered for the use in cyanobacteria are PBAD, P*rha*BAD and P*van*. All of them are controlled by a separately expressed transcription factor. The *E. coli*-derived **PBAD** is induced in the presence of arabinose and repressed by the transcription factor AraC. It was first used and further characterised in Sce_7942 (Cao et al. 2017; Huang et al. 2016) and subsequently optimised for applications in Sy_6803 (Immethun et al. 2017). In Sce_7942, PBAD yielded a relatively high activity (approximately 50% of Ptrc) and low expression in the absence of the inducer, resulting in a 3500-fold dynamic range (Cao et al. 2017). In contrast to Ptrc, a homogeneous and linear expression of the reporter gene in response to arabinose was reported for this promoter. Tight repression of PBAD in the absence of arabinose was also observed in Sy_6803. However, expression levels in this strain were rather low (Immethun et al. 2017). Similar results were obtained for the rhamnose-induced and RhaS-regulated promoter **P*rha*BAD** from *E. coli*. Most recently, this expression system was successfully used in Sy_6803, yielding moderate activity, tight expression control and a 6000-fold dynamic range (Kelly et al. 2018). Less efficient performance was observed for **P*van***, a vanillate-responsive and VanR-repressed promoter from *Corynebacterium glutamicum*. Tight control but only low expression levels were achieved in Sce_7942, while expression completely failed in Sy_6803, A_7120, L_BL0902 and Sy_WHSyn (Taton et al. 2017). Moreover, the usability of a 3-*oxo*-hexanoyl homoserine lactone-responsive system (i.e. the LuxRI system and the corresponding **P*luxRI*** from *Vibro fischeri*) was tested in Sy_6803; however, only poor product formation was observed (Guerrero et al. 2012).

Further, interesting environmental sensors such as light-dependent and O_2_-responsive promoters are used as heterologous induction systems in cyanobacteria. Besides the commonly used plant-derived light-inducible P*psbA* promoters described in the previous section, a novel, sophisticated, darkness-induced regulatory circuit is available for cyanobacteria (Immethun et al. 2017). The system is based on the artificial transmembrane protein Cph8, a hybrid of the native light sensor protein Cph1 from Sy_6803 and the kinase EnvZ from *E. coli*. In response to darkness, Cph1 phosphorylates EnvZ. The latter then phosphorylates the *E. coli*-derived transcription factor OmpR, which finally activates transcription from the promoter **P*ompC***. The system was established in *E. coli* (Levskaya et al. 2005) and engineered for the use in Sy_6803 by Immethun and co-workers (Immethun et al. 2017). In Sy_6803, this system was demonstrated to drive expression of the reporter gene *eyfp* in the dark, while fluorescence was completely absent under low light conditions (i.e. 50 μmol photons m^−2^ s^−1^). Product yields are conspicuously low compared to other expression systems. However, the P*ompC*-based regulatory circuit might be a useful tool to control processes that require dark conditions, such as H_2_ production (Immethun et al. 2017).

Furthermore, the rather newly established O_2_-responsive promoter **PO**_**2**_ should be outlined (Immethun et al. 2016). PO_2_ is activated by the fumarate and nitrate reduction protein FNR under anaerobic conditions in the dark. The system originates from *E. coli*, where the FNR protein controls the expression of several genes during the transition between aerobic and anaerobic growth (Kang et al. 2005). In Sy_6803, PO_2_ yielded moderate expression levels of the flavin-binding fluorescent protein (FbFP), an oxygen-independent reporter, under low O_2_ conditions (Immethun et al. 2016). Only low expression was observed under aerobic conditions. A 28-fold dynamic range of induction was achieved.

PO_2_ was further used to build up a more complex regulatory circuit, which allows tight control of transcription from the *Salmonella typhimurium*-derived promoter **P*SicA*** (Immethun et al. 2016). The concept of the so-called 2-input AND gate is based on the idea to improve the control of target gene expression by the simultaneous application of two different induction systems. P*SicA* is part of the type III secretion system from Salmonella Pathogenicity Island 1 and naturally activated by a complex of the chaperon SicA and the transcription factor InvF (Darwin and Miller 2001). In Sy_6803, SicA* (a mutant version of SicA) was expressed under the control of PO_2_, while invF was transcribed from P*tet*, thus leading to formation of the SicA*–InvF complex and activation of transcription from P*SicA* only upon induction with aTc and in the absence of O_2_ (Immethun et al. 2016). A strong activity (higher expression levels than from PO_2_) and low leakiness were observed for this AND gate in Sy_6803. An approximately 37-fold dynamic range was achieved. Consequently, this regulatory circuit is a suitable tool for expression in Sy_6803 and might also be used in other cyanobacteria.

In summary, the strongest inducible heterologous promoters used in cyanobacteria are P*psbA* and Ptrc, while good induction control was achieved for P*ompC*, P*rha*BAD or Ptrc2O. Promising promoters combining both properties are L03, P*sicA* as well as PBAD and P*tet* for applications in Sce_7942. Anyway, expression levels are slightly lower than for the constitutive expression systems.

### Native, inducible promoters

Native, inducible promoters used for engineering of cyanobacteria encompass metal-inducible promoters, environmental sensor- or metabolic state-dependent promoters and cell type-specific promoters. Metal-inducible promoters often drive the expression of metal efflux pumps or other systems involved in metal homeostasis (García-Domínguez et al. 2000). Metal ions such as Cu^2+^, Ni^2+^, Fe^2+^ or Zn^2+^ are essential for the cellular metabolism, but high concentrations are toxic to the cell (Cavet et al. 2003). Thus, systems for sensing, uptake, storage and excision of metal ions are tightly regulated and highly sensitive to their inducers (Michel et al. 2001; Peca et al. 2008). Widely used cyanobacterial metal-responsive promoters were extensively reviewed by Berla and co-workers in 2013 (Berla et al. 2013). They include systems responding to various metal ions, i.e. Ni^2+^, Zn^2+^, Cu^2+^, Co^2+^, Cd^2+^, Fe^2+^, Fe^3+^, As^3+^ and As^5+^. Common promoters induced by Ni^2+^, Co^2+^ and As^3+^/As^5+^ are **P*nrsB***, **P*coaT*** (or P*coa*) and **P*arsB***, respectively—all controlling the expression of inducer-specific efflux pumps in Sy_6803 (Blasi et al. 2012; Peca et al. 2008, 2007). P*nrsB* was recently found to be the most versatile and useful promoter among several tested native promoters from Sy_6803 because it allows quite good induction control and achieves high expression levels (nearly up to the activity to P*psbA2*) (Englund et al. 2016). This is consistent with previous findings (Blasi et al. 2012). Another promoter controlling the expression of a metal efflux pump is the Zn^2+^-transporting P-type ATPase promoter **P*zia*** from Sy_6803 (Blasi et al. 2012; Peca et al. 2007). A second well-known Zn^2+^-responsive promoter is **P*smt*** from Sce_7942, which controls transcription of a metallothionein gene (Erbe et al. 1996). Recently, this promoter was successfully applied for the expression of dCas9 for CRISPR interference (CRISPRi) in Sce_7942 (Huang et al. 2016). Examples for Fe^2+^- and Fe^3+^-responsive promoters are **P*idiA*** from Sce_7942 and **P*isiAB*** from Sce_7002 or Sy_6803. All of them activate the expression of their target genes in response to iron starvation in their native hosts (Boyanapalli et al. 2007; Kunert et al. 2000; Michel et al. 2001). Moreover, P*isiAB* from Sy_6803 was shown to yield moderate to high expression levels in A_7120, Sce_7942 and L_BL0902, but induction control was rather low (Taton et al. 2014). Furthermore, Cu^2+^-inducible **P*petE*** promoters natively controlling the expression of plastocyanin in A_7120 and Sy_6803 have been applied for engineering purposes in past and recent studies (Buikema and Haselkorn 2001; Englund et al. 2016; Guerrero et al. 2012; Higo et al. 2018a, 2016). Another rather newly described Cu^2+^-responsive promoter is **P*copM*** from Sy_6803. It promotes transcription of a copper-binding protein involved in copper resistance in the presence of its inducer Cu^2+^ (Giner-Lamia et al. 2015). Recently, P*copM* was successfully applied to create a suicide switch in Sy_6803 (Čelešnik et al. 2016). Furthermore, induction of P*copM* by metal ions other than Cu^2+^ was reported (i.e. Zn^2+^, Cd^2+^, Ni^2+^). Such metal-ion cross-reactivity was also described for other metal-inducible promoters (Blasi et al. 2012; Peca et al. 2007). However, induction control by the original inducer metal is usually more efficient and expression levels are higher. In general, induction ranges higher than 100-fold were obtained for several native promoters due to good induction control, but most of them yield relatively low expression levels compared to heterologous systems. The most efficient native systems are P*nrsB*, P*arsB*, P*isiAB* and P*idiA*. For more details on promoter strengths and expression control, refer to Online Resource 1.

Several native promoters respond to environmental conditions and the metabolic state. Light-induced expression systems such as P*psbA2* are described in the ‘Constitutive promoters’ section as they are commonly applied under constant light exposure. Response behaviour to light was investigated for **P*psbA2*** from Sy_6803, which was strongly induced by high light and tightly controlled (Albers and Peebles 2017; Lindberg et al. 2010). In contrast to this, **P*psbA1*** from Sce_7942 showed slightly higher activity upon exposure to low light compared to high light (Nair et al. 2001). Vice versa, **P*psbAIII*** from Sce_7942 was more strongly induced by exposure to high light compared to low light. Interestingly, green light-responsive expression control was reported for **P*cpcG2*** in Sy_6803 (Abe et al. 2014). This promoter is regulated by the green light-sensing histidine kinase CcaS and the cognate response regulator CcaR. Expression from P*cpcG2* was induced under green light (or green and red light), but not under red light illumination. The expression strength of this promoter was very low compared to Ptrc, yet it could be strikingly improved (up to 30% of Ptrc) by insertion of a Shine–Dalgarno-like sequence derived from the *cpcB* gene and overexpression of CcaR. However, expression from this version was leaky. The highest dynamic range (i.e. 15-fold induction range) and relatively tight expression control was observed for the modified version without overexpression of CcaR (i.e. P*cpcG2*-SD), whereby the activity was slightly improved compared to the native version (Abe et al. 2014). Native P*cpcG2* was successfully applied for the expression of T4 bacteriophage lysis genes (Miyake et al. 2014).

Further environment-sensing systems respond to CO_2_ limitation, i.e. **P*cmpA*** and **P*sbtA*** from Sy_6803 (Liu et al. 2011; McGinn et al. 2003). Both of them are tightly controlled and yield high dynamic ranges (1400-fold and 800-fold, respectively). A more recent environment-responsive expression system is **P*phoA*** from Sce_7942 (Taton et al. 2014). This promoter is repressed by inorganic phosphorus and thus can be induced by continuous phosphorus limitation. The activity of P*phoA* was investigated in Sce_7942, Sy_6803, A_7120 and L_BL0902. Moderate to high expression levels were reported for all strains, while satisfying induction control was only observed in Sce_7942 and L_BL0902 (Taton et al. 2014).

Furthermore, cyanobacteria strictly respond to the availability of nitrogen sources. The nitrate reductase promoters **P*nirA*** (or P*nir*) from A_7120, Sce_7942 and Sy_6803 are induced by nitrate and repressed by ammonium and have been widely used for engineering purposes (Camsund et al. 2014; Desplancq et al. 2005; Ivanikova et al. 2005). They usually yield tight expression control and moderate to strong activities. Lately, also a promoter induced by nitrate-starvation was characterised in Sy_6803, i.e. **P*sigE***, the promoter for the RNA polymerase group 2 sigma factor SigE (Immethun et al. 2017). P*sigE* showed the strongest expression among the five NtcA-regulated promoters tested in this study, yet expression levels in the presence of nitrate were very high. Anyway, a 30-fold dynamic range was achieved.

Further native promoters associated with the nitrogen metabolism are cell type-specific promoters. Several filamentous cyanobacteria such as A_7120 are capable of differentiating some vegetative cells into heterocysts under nitrogen starvation conditions (Stewart et al. 1969). These heterocysts are cell types specialised for N_2_-fixation and provide a microaerobic environment, which is essential to protect the O_2_-sensitive nitrogenase, i.e. the enzyme performing N_2_-fixation (Fay 1992). Such cell type-specific promoters are useful tools to express O_2_-sensitive target products in heterocyst-forming strains. The most prominent examples of heterocyst-specific promoters are the promoters **P*nifB*** and **P*nifB1*** controlling expression of the nitrogenase genes in A_7120 (Mulligan and Haselkorn 1989) and Av_29413 (Haselkorn and Buikema 1992), respectively. Another heterocyst-specific promoter from A_7120 is **P*coxBII***, which drives the expression of subunit II of a cytochrome *c* oxidase (Jones and Haselkorn 2002). Cell type-specific expression control was demonstrated for all three promoters via reporter gene analysis (Thiel et al. 1995; Ungerer et al. 2010; Wang and Xu 2005). Moreover, P*nifB* and P*coxBII* were recently used to control the expression of O_2_-sensitive enzymes of the 1-butanol synthetic pathway from the anaerobe bacterium *Clostridium acetobutylicum*, thereby finally allowing heterologous 1-butanol production in heterocysts of A_7120 (Higo and Ehira 2019). The expression from P*nifB* and P*coxBII* was additionally regulated by exogenous riboswitches, i.e. a theophylline- and a 2-aminopurine (2-AP)-responsive riboswitch, respectively. Quantities of the 1-butanol produced in the obtained strain were 5-fold higher than those produced with O_2_-tolerant enzymes in Sce_7942 (Higo and Ehira 2019). The usability of the riboswitches and further RNA-based tools as control elements for cell type-specific expression in A_7120 was analysed in a preceding study (Higo et al. 2018b). Heterocyst-specific and vegetative cell-specific expression was demonstrated using the native promoters P*nifB* and **P*rbcL***, respectively. P*rbcL* from A_7120 had already been demonstrated to promote vegetative cell-specific expression in an earlier study (Wang and Xu 2005). Another vegetative cell-specific promoter is **P*nifB2*** from Av_29413. It controls the expression of a second nitrogenase in Av_29413, which, in contrast to the above-described enzyme controlled by P*nifB1*, is exclusively produced in vegetative cells during anaerobic conditions (Thiel et al. 1995; Vernon et al. 2017). Moreover, an expression system specific for prospective and immature heterocysts was reported in A_7120 (Muro-Pastor 2014). The promoter of the nitrogen stress inducible RNA1, namely **P*nisR1***, drives transcription only in early stages of heterocyst formation, but not in mature heterocysts. It belongs to the family of DIF+ class promoters and is the shortest native promoter (70 nt long) controlling heterocyst-specific expression (Mitschke et al. 2011). Most recently, an even shorter promoter (48 nt long) specific for the expression in mature heterocysts was synthetically generated based on regulatory elements of the DIF+ class promoters (Wegelius et al. 2018). This promoter, termed **PsynDIF**, allowed tight control of reporter gene expression in a cell type-specific manner in Np_29,133. Strong expression yields were observed and a 10-fold dynamic range was achieved. Consequently, PsynDIF substantially increases the set of tools for cell type-specific expression in heterocyst-forming cyanobacteria.

Cell type-specific promoters and promoters based on interesting induction systems such as P*phoA*, P*cpcG2*, P*cmpA* or P*sbtA* should be kept in mind as promising systems for gene expression control. Further important native, inducible promoters are some metal-inducible promoters, P*petE* and P*nir*. However, overall expression yields obtained from those systems lag behind those of heterologous, inducible and constitutive expression systems.

## Regulatory RNAs

Regulatory RNAs used for expression control in cyanobacteria encompass riboswitches, riboregulators and further sRNA-based tools. For details on the respective regulatory elements, refer to Online Resource 1.

### Riboswitches

**Fig. 1:**
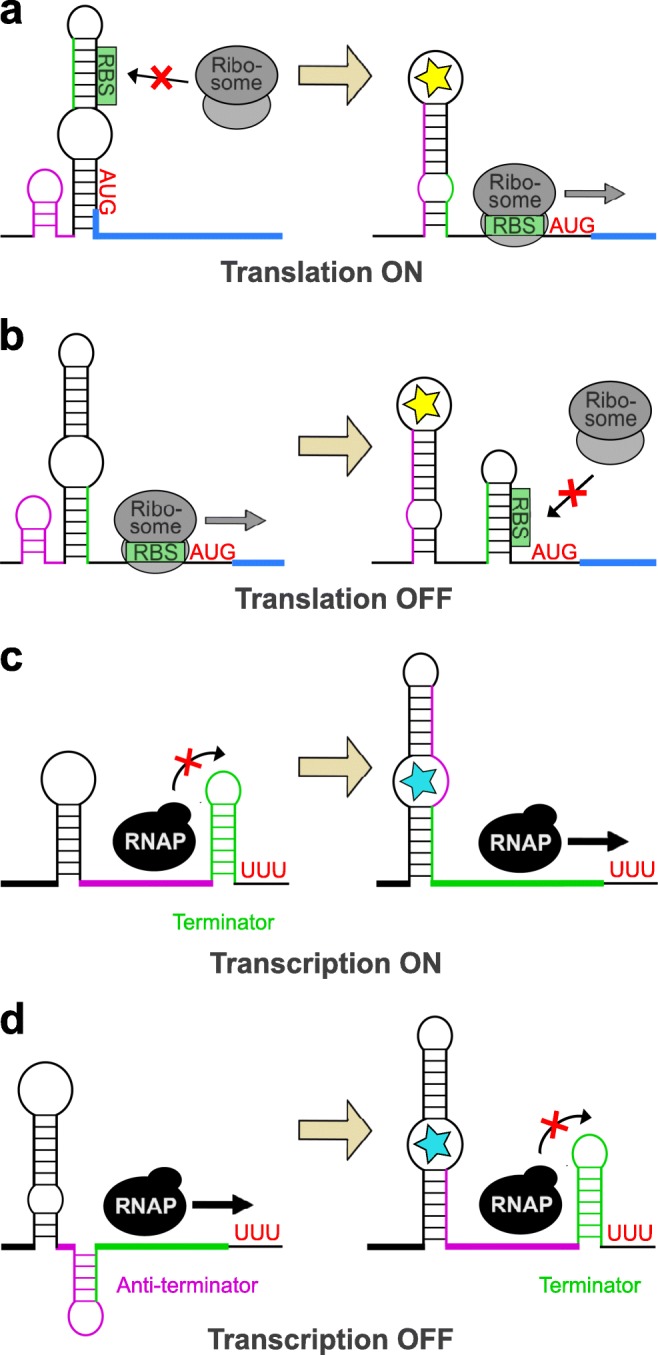
Operation mode of transcriptional and translational riboswitches. Conformational changes of riboswitches in the absence (left) and presence (right) of a specific ligand (yellow or cyan star) result in a transcriptional or translational ON/OFF switch of gene expression. **a** Translational ON riboswitch. In the absence of the ligand, the ribosome binding site (RBS) is sequestered by the riboswitch. Upon binding of the ligand, a conformation change makes the RBS accessible to ribosomes, thus enabling translation. **b** Translational OFF riboswitch. In the absence of the ligand, the RBS is accessible to ribosomes, consequently allowing translation. Upon binding of the ligand and a conformational change, the RBS becomes inaccessible for ribosomes and the translation is switched off. **c** Transcriptional ON riboswitch. In the absence of the ligand, transcription performed by the RNA polymerase (RNAP) is terminated by a hairpin structure (terminator). Upon binding of the ligand and conformational changes, the hairpin unravels, thereby allowing progression of the RNAP and full-length transcription of the target gene. **d** Transcriptional OFF riboswitch. In the absence of the ligand, the riboswitch forms an anti-terminator hairpin, consequently allowing full-length transcription of the target gene. Upon binding of the ligand and conformational changes of the riboswitch, a terminator hairpin is formed and the RNAP is dissociated, thus resulting in an offset of transcription

Riboswitches are *cis*-regulatory RNA elements on mRNAs, usually located in the 5′ untranslated regions (UTR). In response to binding of a specific ligand, i.e. metabolite or signal molecule, they undergo a conformational change, which finally results in an ON/OFF switch of gene expression. Riboswitches are composed of two domains: an aptamer and an expression platform. The aptamer is responsible for specific ligand-binding, while the expression platform mediates interference with the gene expression machinery (Garst et al. 2011). Riboswitches can modulate gene expression in a transcriptional or translational manner. Translational riboswitches provoke an ON/OFF switch of translation by altering the accessibility of the RBS, while transcriptional riboswitches allow or block the formation of a terminating hairpin structure. Both transcriptional and translational riboswitches have either an activating (i.e. ON) or repressing (i.e. OFF) effect in response to ligand binding. The possible modes of regulation by a riboswitch are illustrated in Fig. 1.

Most riboswitches used in cyanobacteria are induced by theophylline. The best-characterised system is a translational ON riboswitch termed **Riboswitch F** (also referred to as Riboswitch E*). Riboswitch F is one of a set of six synthetic, theophylline-responsive riboswitches, **Riboswitch A**, **B**, **C**, **D**, **E** and F, developed for applications in various Gram-positive and Gram-negative bacteria (Topp et al. 2010). All six riboswitches were used for reporter gene studies in various cyanobacterial strains, i.e. Sce_7942, A_7120, L_BL0902 and Sy_WHSyn (Ma et al. 2014; Nakahira et al. 2013). Slightly leaky to tight induction control in response to theophylline was achieved for expression from the constitutive promoter PconII in all strains (Ma et al. 2014). The dynamic ranges differed between the riboswitches and strains, but overall they were higher in Sce_7942 and L_LB0902 than in A_7120 and Sy_WHSyn. The highest expression levels after induction were achieved with Riboswitch E, which however also featured the highest expression in the absence of the inducer. Tight expression control and the best induction efficiency were observed for Riboswitch F, with a 25-fold dynamic range in Sce_7942. An even higher dynamic range (190-fold) was reported for Riboswitch F-regulated expression from Ptrc in Sce_7942 (Nakahira et al. 2013). Consequently, Riboswitch F emerged as the most efficient candidate of the investigated, synthetic theophylline riboswitches and was applied for induction control in further studies. Moreover, its functionality in Sy_6803 was demonstrated by reporter gene analysis (Ohbayashi et al. 2016). In this strain, Riboswitch F was recently used to convert a repressive sRNA tool based on paired termini antisense RNAs (PTRNAs, see ‘Riboregulators and small RNAs’ section) into an inducing system (Sun et al. 2018b). As another example, Riboswitch F was applied as an additional control element for *dCas9* expression in order to improve the dynamic range of *glnA* repression in a CRISPRi study (Higo et al. 2018a). The goal of this study was to fine-tune intracellular glutamine synthase A (GlnA) levels and to enhance ammonia production in A_7120. Due to leaky expression from Ptrc, dCas9 was continuously produced in the absence of additional control elements, which resulted in constant repression of the essential *glnA* gene and cell death in the absence of nitrogen sources. Riboswitch-based regulation allowed tight control of dCas9 production and *glnA* repression (Higo et al. 2018a).

In the same study, also other RNA-based tools and riboswitches were successfully applied for the regulation of *dCas9* expression (Higo et al. 2018a). Among them is another theophylline-inducible riboswitch, namely the transcriptional ON riboswitch *theo*/pbuE*. This riboswitch was created by chimeric fusion of the engineered expression platform derived from the adenine-responsive *pbuE* riboswitch from *Bacillus subtilis* with a theophylline-responsive aptamer (Ceres et al. 2013). The artificial theophylline-responsive riboswitch *theo*/pbuE* as well as the *B. subtilis*-derived, adenine-responsive riboswitch *pbuE*/pbuE* were first tested for the control of reporter gene expression and subsequently applied for cell type-specific expression in A_7120 (Higo et al. 2018b). Both riboswitches allowed specific induction control in response to their inducers. For *pbuE*/pbuE*, the expression output was higher for induction with 2-AP compared to its analogue adenine, but in either case the expression was leaky. The efficiency of induction control was conspicuously better for *theo*/pbuE*. Relatively tight control and a 25-fold dynamic range were achieved. Both riboswitches allowed spatio-temporal gene induction in heterocysts and vegetative cells (Higo et al. 2018b) and were successfully applied for the regulation of 1-butanol-production in A_7120, while expression control using the above-described Riboswitch F was not successful (Higo and Ehira 2019).

Two further transcriptional riboswitches used in cyanobacteria are a native cobalamin riboswitch and the adenine/2-AP-responsive *xpt*(C74U)/*metW*—both of them are transcriptional OFF riboswitches. Just like *pbuE*/pbuE*, ***xpt*(C74U)/*metW*** originates from *B. subtilis* and was recently used for expression control in A_7120 (Higo et al. 2017). It was applied as a regulatory switch for *tetR* expression, which finally allowed tight control of the expression of a reporter gene produced from the TetR-repressive L03 promoter. Functionality of *xpt*(C74U)/*metW* in A_7120 was indirectly proven. Notably, also the usability of a cyanobacteria-derived riboswitch, namely the **Cobalamin Riboswitch** from Sce_73,109, was tested (Pérez et al. 2016). The riboswitch regulates the production of the cobalamin (i.e. vitamin B^12^)-dependent version of the Methionine Synthase (MetH) in Sce_73109 and was analysed in Sce_7002 via reporter gene assay. Slightly leaky expression in response to cobalamin was reported and a 6-fold dynamic range was achieved (Pérez et al. 2016). Overall, the dynamic ranges obtained from the application of riboswitches are rather low. Nevertheless, the reviewed studies clearly demonstrate that riboswitches can be regulated independently from the promoter and thus are promising tools for improving gene expression control in cyanobacteria.

### *Trans*-RNA-based tools

**Fig. 2:**
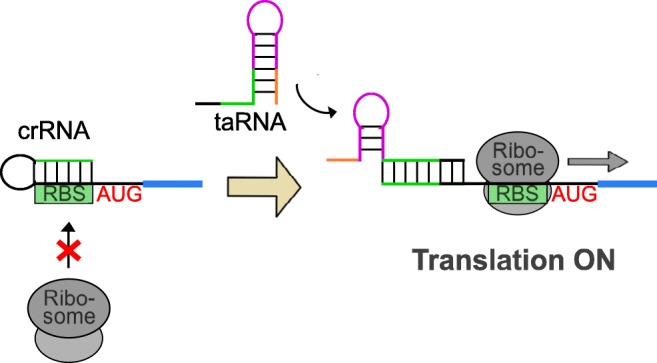
Operation mode of a *trans*-acting, translational ON riboregulator. Conformational change of a *cis*-repressed RNA (crRNA) in the presence (right) compared to the absence (left) of a *trans*-activating RNA (taRNA) results in a translational ON switch of gene expression. In the absence of the taRNA, the 5′UTR of the mRNA forms a repressive *cis*-repressed RNA hairpin structure, which sequesters the ribosome binding site (RBS), thereby preventing translation initiation. Structural changes of *cis*-repressed RNA upon binding of the taRNA lead to exposure of the RBS and thus allow the onset of translation

In addition to riboswitches, riboregulators acting in a *trans*-mode can be applied for targeted gene expression control. They function in a similar way as riboswitches. A *cis*-element on the mRNA undergoes structural changes, in this case in response to the binding of a *trans*-acting RNA. This results in modulation of the accessibility of regulatory regions and gene expression control. The common mode of such a riboregulator is a translation ON mode (see Fig. 2). A typical example was established in *E. coli* by Isaacs and co-workers (Isaacs et al. 2004). The system is based on the action of two RNAs: a *cis*-repressed RNA and a *trans*-activating (taRNA). In the absence of the taRNA, the RBS is occluded by an internal stem-loop structure formed by the *cis*-repressed RNA (namely crR12), while conformational changes upon binding of the taRNA (namely taR12) lead to exposure of the RBS (Isaacs et al. 2004). In 2014, this riboregulator was engineered for applications in Sy_6803 by introducing the strong RBS* into crR12 (Abe et al. 2014). The obtained riboregulator taR*2/crR*2 allowed low leaky expression control when the taRNA was expressed from the arabinose-inducible promoter PBAD; however, the product yield was relatively low. Expression efficiency of this riboregulator could be improved by fusion of the taRNA to *E. coli* scaffold RNAs, which contain a binding site for the RNA chaperon Hfq and a rho-independent transcription terminator sequence (Sakai et al. 2015). The best result (a 19-fold dynamic range, 2.5-fold higher than for taR*2) was achieved for a slightly modified version of taR*2/crR*2 fused to the scaffold RNA MicF (termed taR*2-MicF M7.4) in a Sy_6803 strain expressing the *E. coli*-derived Hfq. An even higher ON–OFF ratio of taR*2/crR*2 (up to 50-fold) was reported for the regulation of the chromosomal gene encoding the Abr-like transcription factor cyAbrB2 (Ueno et al. 2017). Moreover, in a recent study the dynamic range was increased up to 78-fold by optimisation of the intra- and intermolecular riboswitch hybridisation (Sakamoto et al. 2018). Highest expression levels combined with very low baseline expression in the uninduced state were obtained for a version termed taR*4/cr*4-AA. These studies demonstrate that structural fine-tuning and stabilisation of the taRNA are powerful strategies to improve the performance of riboregulators.

Besides its application in Sy_6803, the riboregulator taR*2/crR*2 was also used as a regulatory element for heterocyst-specific expression in A_7120 (Higo et al. 2018b). The usability of taR*2/crR*2 was compared to three similar *trans*-sRNA-based tools: the **toehold** switch (Green et al. 2014), small-transcription activating RNAs (**STARs**) (Chappell et al. 2015) and **STARs (dual)** (Westbrook and Lucks 2017), which act as activators of transcription, translation or both, respectively. The *cis*-element was placed upstream of a *lacZ* reporter gene and fused with P*nifB*, while the *trans*-acting RNA was expressed under the control of the aTc-inducible L03 promoter (Higo et al. 2018b). Leaky expression in heterocysts in the absence of aTc was observed for STARs and STARs (dual), while rather tight induction control was possible for toehold and taR*2/crR*2. Notably, tight control was achieved for the STARs (dual) system in another study (Higo et al. 2018a).

Another *trans*-sRNA-based tool is the *E. coli*-derived **IS10** (Kittle et al. 1989). Like a classical riboregulator, this system consists of a *cis*-element on the 5′UTR of the mRNA, the RNA-IN, and a separately expressed antisense RNA, the RNA-OUT. The RNA-OUT folds into a hairpin, yet, upon binding to the RNA-IN, it unfolds and base-pairs with the RBS on the RNA-IN, thus preventing the onset of translation. In Sce_7002, the IS10 system allowed tightly regulated induction and resulted in 70% attenuation of the target gene expression (Zess et al. 2016). Even 90% attenuation of target gene expression was achieved in *E. coli* (Mutalik et al. 2012).

Two further sRNA regulatory tools have recently been established in Sy_6803; both were originally developed in *E. coli* (Sun et al. 2018b). One tool is based on paired termini *trans*-sRNAs (**PTRNAs**) (Nakashima et al. 2006), which works similar to the IS10 system. The PTRNA contains two short inverted repeats, which base pair, thus resulting in the formation of a hairpin. The loop region of the hairpin contains a target-specific antisense sequence, which hybridises with the target gene, resulting in inhibition of translation and post-transcriptional mRNA degradation. The other tool, namely **Hfq/MicC**, makes use of the scaffold RNA MicC, which is fused to a target-specific antisense sequence and recognised by the Hfq chaperon (Na et al. 2013). Via target-specific binding of MicC, Hfq is directed to the final location and causes mRNA degradation by the recruitment of the major endo-ribonuclease RNase E. Both tools allowed an approximately 90% repression of target gene expression (Sun et al. 2018b). In both cases however, induction control was leaky.

Furthermore, the expression of **target-specific antisense RNAs** (asRNAs) is a simple strategy for gene expression control. Higo and co-workers applied numerous artificial TetR/*tetR*-specific RNAs to regulate gene expression in A_7120, i.e. asRNAs specific for *tetR*-P*petE* and *tetR*-P*nirA*, a TetR-aptamer (with and without a tRNA scaffold) and a TetR inducing peptide (i.e. Tip-TrxAss) (Higo et al. 2017). An impact on the final expression output was observed for the two gene-specific asRNAs and the protein-binding TetR-aptamer stabilised by a tRNA scaffold. Moreover, expression of the sigma factor SigJ was successfully down-regulated by the expression of a *sigJ*-specific asRNA in A_7120 (Srivastava et al. 2017). However, all target-specific RNA-based tools had a relatively low regulatory effect and need to be improved for more sophisticated applications.

Like riboswitches, riboregulators and sRNA-based tools can be applied as additional control elements, regulated independently from the promoter of the target gene. They allow even more efficient gene expression control compared to riboswitches and are promising tools for future applications.

## Multicomponent regulatory systems and regulatory circuits

The application of multiple components for the regulation of target gene expression proved to be a suitable strategy to improve expression efficiency and induction control. For some promoters, separately produced activators or repressors such as LacI, TetR, AraC, RhaS, VanR or FNR are required for regulation control. Fine-tuning of the levels of these transcription factors might allow optimisation of target gene expression. This fine-tuning can be achieved by the application of additional regulatory elements or by variation of the chosen promoter. As an example, very divergent results were obtained for the promoter L03 in studies using different promoters for the expression of TetR (Higo et al. 2016, 2017; Huang and Lindblad 2013).

Moreover, regulatory circuits depending on the action of multiple regulatory proteins have been presented in the previous sections of this article. The chimeric dark-sensor Cph8 transfers the environmental signal to the transcription factor OmpR, which finally activates expression from P*ompC* (Immethun et al. 2017). Another example is the 2-input AND gate based on the transcription factor InvF and the chaperon SicA*, which together act on the induction from P*SicA* (Immethun et al. 2016). Both systems are described in more detail in the ‘Heterologous, inducible promoters’ section.

Furthermore, riboswitches and riboregulators are powerful tools for fine-tuning of expression levels and for improving induction control, as both sorts of RNA tools can be regulated independently of the target promoter (by the addition of the ligand or expression of the taRNA, respectively). For both tools, the dynamic range is relatively low compared to promoters, yet it could be strikingly increased by slight alterations of the sequence or structure. Advanced circuit systems including riboswitches were created by Taton and co-workers (Taton et al. 2017). The so called NOT gates are based on the idea to produce an OFF output in response to an ON signal by the application of an inducible riboswitch, which controls the expression of a transcription repressor such as LacI and VanR. Similar systems and further complex multicomponent studies including riboswitches or riboregulators have been published (Higo and Ehira 2019; Higo et al. 2018b, 2017, 2016) and were discussed in more detail in the previous sections.

## Summary and conclusion

While constitutive expression works quite well in cyanobacteria, several heterologous or native, inducible promoter systems suffer either from leakiness or from a low expression output. Anyway, in recent years, existing systems have been improved and new promoters have been discovered or engineered for cyanobacteria. Parameters such as the promoter length, slight variations of the sequence and promoter elements (i.e. the −10 and −35 regions, the interspace between them, operators and the RBS) were identified as crucial factors for the promoter efficiency and hold the potential for further optimisation of expression systems for enhanced applications in cyanobacteria. Moreover, alternative strategies for expression control in cyanobacteria have been established. Riboswitches and riboregulators can be regulated independently of the target promoter and emerged as powerful tools for fine-tuning of expression levels and enhancing induction control. Further sRNA-based approaches and target-specific sRNAs essentially increase the toolset of RNA-based regulatory systems. The technological progress on genome-wide screening and transcriptome analysis will lead to the identification of further promising candidates of native regulatory RNAs. Another upcoming strategy for the regulation of gene expression is the generation of genetic circuits via the combination of different regulatory modules. More work in this field will be necessary to further advance efficient and tightly controlled gene expression in cyanobacteria.
